# Quinium Salts

**Published:** 1883-11-20

**Authors:** R. Rother


### QUINIUM SALTS.

By R. ROTHER, M. D.

The quinium salts found in the general market, excepting the sulphate, are very much less in demand than this great staple salt, probably because it appeared earliest in the field and so became the dominant form. Had the chloride, instead of the sulphate, been first presented its introduction would have followed as a matter of course. It is, indeed, to be regretted that this was not the case, since the chloride has more specially valuable qualities than are possessed by any other quinic salt. Containing a greater percentage of quinine it is at the same time more soluble in either alcohol or water than any other normal quinium salt; and, although far more soluble than the normal sulphate, it is much less bitter and less persistently bitter than this. Another advantage is that owing to its greater solubility all other less soluble salts of quinine can be prepared from it by double decomposition. Whilst being the staple form it could also be the cheapest salt of the market, and hence all other varieties prepared from it would be correspondingly less costly than now.

For preparing the various quinium salts from the sulphate, two methods are in use: One consists in precipitating the base from the acid sulphate by means of caustic alkali, and dissolving it in the acid of which the salt is desired. By the other method either the normal or the acid sulphate is decomposed by the barium salt of the acid whose quinium compound is to be obtained. In certain cases other processes or modifications of the foregoing may be employed with advantage.

The first general method is rarely desirable, and the use of the barium salts although frequently directed is not often resorted to, the corresponding calcium salt with alcohol being employed in most cases with superior effect. By one method strong alcohol is employed and the entire precipitation of the by-product is aimed at. In the second, only sufficient alcohol is added after the completion of the reaction to effect the solution of the generated quinium salt in the least volume of aqueous menstruum.

Of several processes for producing quinium chloride the best is afforded by taking advantage of its almost total insolubility in a saturated solution of sodium chloride. When any convenient amount of quinium sulphate is mixed with a hot filtered saturated solution of sodium chloride, the quinium chloride is precipitated as a crystalline magma which rapidly agglutinates, and, on cooling, forms a compact friable mass. The liquid is poured away from the residue, which is washed from the sodium sulphate generated by heating several more times with sodium chloride solution. The quinium chloride may be crystallized from hot water in the usual manner.

Quinium hypophosphite is usually made by dissolving the free base in hypophosphorus acid and crystallizing. The best result is obtained by dissolving 170 parts of calcium hypophosphite in 15,-

000 parts of water, heating the solution and adding 872 parts of quinium sulphate, filtering after the calcium sulphate has subsided and setting the solution aside to crystallize.

The union of tannin with quinine, the so-called tannate, in view of its medicinal inferiority and excessive cost, is yet considerably used, probably on account of its lack of bitterness. This salt, if salt the ordinary article may be called, contains a very low, in fact the very lowest percentage of quinine, and is also one of the most insoluble compounds of this base.

Tannin has a varied affinity for many substances, and in different degrees for the same substance. By reason of this peculiarity the quinium tannate, or tannolate, as ordinarily prepared, contains a very large excess of tannin compounded with free acid, so that the article is in reality a mixture. Manufacturers, indeed, endeavored to have the largest possible amount of tannin absorbed, deeming such a procedure perfectly legitimate in view of the fact that no recognized and definite standard for comparison exists. The bitterness of the substance, of course, diminishes in proportion to the deficiency of quinine contained in it, and the degree of its envelopment by the inert acid tannolate.

There is a quinium sulpho-tannolate of fairly definite composition which might, with advantage, replace the other so-called tannates. It may be readily prepared by the following formula: Dissolve 322 parts of tannin and 98 parts of potassium acetate in 10,000 parts of water, by the aid of heat, then add 872 parts of quinium sulphate, continue the heat for a few minutes, transfer the precipitate to a filter, and after sufficient washing dry it by exposure to the open air.

Syrup of yerba santa is growing in popularity as a vehicle for quinine in a tasteless form. As ordinarily prepared, it represents one ounce of the leaves in the pint, but a syrup of half this strength would be quite as good for general purposes. The active agent is an acid resin, which generates with quinine a nearly insoluble salt, which is decomposed by the common acids in the free acid resin and soluble quinium salt. The compound of quinine with the resin is a definite salt, and would be an excellent substitute for the indefinite tannolates of the market. It can be readily produced by extracting the leaves with water containing some alcohol and ammonia, and mixing the liquor with quinium sulphate, warming gently, washing the precipitate and drying it by exposure.

Syrup of yerba santa is best prepared by percolating one ounce of the leaves in coarse powder with water containing one drachm of ammonia water and two fluid ounces of alcohol in the pint, until one pint of liquor is obtained, and dissolving twenty-eight troy ounces of sugar in this with gentle heat. This syrup is clear and bright, having a deep brown red color, and slightly bitter but pleasant honey-like taste.

Quinium valerate in two crystalline forms in star-grouped needles, and in plates.

The first kind are obtained from a hot saturated solution by cooling; the second, at a lower temperature by slow evaporation. The first is the most practical form, and most readily produced. Double decomposition is the only practical procedure for preparing the valerate. Two or three methods may be used, but the best process is that by double decomposition between quinium sulphate and calcium valerate in the presence of weak alcohol. This yields the salt chiefly in star crystals. The calcium valerate is generated by the action of valeric acid in aqueous solution on calcium carbonate. The reaction is almost instantly completed with copious effervescence. The formola is as follows: Mix 204 parts of valeric acid with 5,000 parts of water, add 100 parts of calcium carbonate, and when effervescence has ceased and a clear solution has resulted, add 2.500 parts of alcohol and 872 parts of quinium sulphate. Now heat the mixture until decomposition is complete; filter whilst hot, and rinse the residue of calcium sulphate with a little alcohol or weak alcohol, and set the filtrate aside to crystallize. Collect the crystals on a filter, drain, and dry in the open air. The drained liquor on evaporation will yield an additional crop of crystals.—*Abridged from an Article by R. Rother, in the American Journal of Pharmacy.— Va. Med. Monthly.*

